# A Treatise on Obstetrics

**Published:** 1887-06

**Authors:** 


A PRACTICAL TREATISE ON OBSTETRICS. *Vol. I. (4 vols.), Anatomy of the Internal and External Genitals, Physiological Phenomena (Menstruation and Fecundation.) By* A. CHARPENTIER, M.D., *Paris. Illustrated with lithographic plates and wood engravings. This is also Vol. I. of the "Cyclopædia of Obstetrics and Gynæcology" (12 vols.), issued monthly during 1887. New York:* WILLIAM WOOD & COMPANY. *Chicago:* W. T. KEENER.

The publishers are to be congratulated upon their abandonment of a policy which compelled the purchase of books which in many cases were not wanted, in order to obtain others which were desired. Every volume of this series promises to be desirable, and the enterprise which secures the publication of the "*Cyclopædia*" merits commendation.

Anything like a critical review by topics, of even only a part of Charpentier's work on obstetrics, which this volume is, is of course not to be attempted in the limits of a mere book notice, and hence general statements must suffice.

This translation is made under the supervision of, and edited by, Egbert H. Grandin, M.D., and both duties have been well performed. The section in embryology in the original treatise has been replaced by a condensation of the contribution on that subject by Professor Milnes Marshall to Barnes' treatise on obstetrics.

In the many points in which either general opinion has changed since the original treatise was written (1882), or in which Charpentier's views conflict with those generally held in America, the editor has added, in brackets, the necessary statements. This has been done in a very satisfactory manner, as, for instance, the correction on pages 453 and 454, of the author's gross misrepresentation of Credé's method of delivering the placenta.

The colored lithographic plates are quite satisfactory, but the very numerous wood-cuts are poor in every way, and particularly in the explanatory letters about the margin of the cut, which are in a great many cases illegible. The low price of the books in the series is not a sufficient excuse for this fault, which is a grave one, and which ought to be corrected in subsequent editions. The printing and binding of this volume are very fair, and the size is very convenient. E. W.

